# Experimental Characterisation and Finite Element Modelling of Polyamide-11 Fabricated via Multi Jet Fusion

**DOI:** 10.3390/polym18192399

**Published:** 2026-10-01

**Authors:** Ryan Blakis, Mladenko Kajtaz

**Affiliations:** RMIT Centre for Additive Manufacturing, School of Engineering, RMIT University, Melbourne, VIC 3000, Australia; ryan.blakis@rmit.edu.au

**Keywords:** Multi Jet Fusion, polyamide 11, finite element analysis, material model selection, material model calibration, tension–compression asymmetry, TPMS gyroid, build orientation

## Abstract

HP Multi Jet Fusion (MJF) polyamide-11 (PA11) was characterised experimentally and used to calibrate finite element material models for the quasi-static regime. Characterisation covered tension in six build orientations and compression in three. Validation used threepoint bending at XY and YX, and a vertically aligned triply periodic minimal surface (TPMS) gyroid. The flat XY orientation was the anisotropy extreme, carrying a tension–compression yield asymmetry of 30.2% that fell to 14.3% at YX and was negligible at XZ. The modulus asymmetry ran the opposite way, 7% at XY against 20% to 27% elsewhere. Elastic-plastic and asymmetric models were calibrated at the representative YX basis, with XY as the constraining case. Single-stress-state fits deviated by up to 7.8% in the other branch, and the tabular tension-only fit failed outright in compression at YX. At YX the fits carrying both branches held both stress states, the asymmetric formulation trading compressive accuracy for tensile, and at XY it alone held both. In three-point bending the asymmetric YX model matched the experiment, while the XY coupons exceeded both XY-calibrated models, an unexplained flexure elevation. In the gyroid crush the leading YX models reproduced the three-phase response, slightly over-predicting the plateau. YX is recommended as the calibration basis, with the combined-hardening elastic-plastic fit as the general-purpose model. The asymmetric formulation should be considered where bending stiffness governs, on the strength of an empirical rather than mechanistic agreement.

## 1. Introduction

Additive manufacturing (AM), also commonly referred to as 3D printing (3DP), is a manufacturing method that constructs components layer-by-layer based on a digital part file [1]. AM reduces waste, overcomes numerous traditional geometry constraints, and removes the requirement for costly tooling [2]. The method is especially advantageous in industries such as the medical sector, which manufacture complex and highly customised low-volume products [3]. Although the method was developed several decades ago, the mechanical properties of AM components limited its use primarily to rapid prototyping [4]. However, gradual advancements in additive methods and materials have now made it possible to produce end-use functional products [5].

One such method is MJF, a type of powder bed fusion developed by HP [6]. The process begins by spreading a layer of build powder over the designated print area. A fusing agent is applied to specific areas where the powder is intended to solidify. At the same time, a detailing agent is applied around the edges of the part being printed to achieve sharper and more defined surface finishes. The build area is exposed to infrared radiation (IR) which is absorbed by the fusing agent, solidifying the intended areas and leaving the other areas in powder form. This process is repeated until part fabrication is complete. In comparison to selective laser sintering (SLS), another powder bed fusion method, MJF supplies a planar infrared energy input across the whole build area rather than scanning line by line with a laser. This gives a geometry-independent layer processing time of approximately 10 s and a substantially higher build rate [7].

Since its release in 2016, research studies have been conducted on MJF polyamide-12 (PA12) and PA11. Polyamide 11 is reported to exhibit enhanced mechanical properties, including elongation-at-break, impact resistance, and ductility, in comparison to PA12 [8]. Studies have comprehensively investigated both the powder properties [9,10,11] and the characteristics of resulting manufactured components [10,11,12,13]. Mechanical properties are observed to be influenced by build parameters [10,12,13], as well as powder refresh ratios [11]. The strongest of these influences is build orientation, a common theme among AM research. In the context of MJF, some claim there is an absence of significant build orientation-related anisotropy for some mechanical properties like tensile strength and modulus [14,15]. However, the existing literature for both PA11 and PA12 provides contradictory findings in this regard [13,16,17,18]. In a comprehensive study of MJF PA11, Lee et al. (2020) [13] observed noticeable variations in tensile modulus, strength and elongation at break. The authors found that tensile specimens oriented at a 45° incline to the build plate displayed superior performance for all characterised mechanical properties. However, the same study found that compressive mechanical properties were less sensitive to build orientation. The authors proposed that the improved tensile performance in the 45° incline build orientation simultaneously avoided negative influences of poor intra-layer powder packing in the horizontal plane and oriented the intra-layer interface against the load direction. In similar work conducted on PA12, Lee and Kajtaz (2022) [18] extended specimen characterisation to encompass three additional build orientations, which were rotated 90° about the IR sweep axis. Although anisotropy was still present in the mechanical properties of the rotated specimens, it was reduced in comparison to the non-rotated specimens. These results supported the thesis that an increase in IR exposure, the sintering mechanism, would promote more thorough powder fusion and thus improve mechanical performance. These additional build orientations are yet to be investigated for MJF PA11.

The geometric freedom of AM is complemented by computational design, in which component geometries are optimised using tools such as finite element analysis (FEA). Effective use of FEA requires accurate modelling of the material behaviour. A range of material models has been applied to MJF PA11 and PA12, including the linear elastic (LE), elastic plastic with isotropic hardening (EPI) and elastic plastic with combined hardening (EPC) models [18,19,20,21]. Kajtaz and Lee (2019) [19] compared these Abaqus-native models [22] and found the EPC model to perform best in three-point bending. Similar results were reported for PA12 [18]. The simpler LE model has also performed well for PA11 under crushing [21]. More recently, Perez-Barcenilla et al. (2024) [23] formulated a transversely isotropic constitutive model for MJF PA12, MJF lattices have been characterised against analytical scaling laws [24], and bonded components have been simulated [25]. These models are calibrated either to a single stress state or to a compromise between tension and compression, and none of them represents the asymmetry between the two branches. Whether that simplification is adequate has not been established. Lattice crushing introduces nonlinear geometry, contact and structural instability. Where MJF polyamide lattices have been simulated, the material model was calibrated in tension alone [20] or was linear elastic [21], so a model calibrated across both stress states has not been evaluated under those conditions.

The rotated build orientations that reduced anisotropy in PA12 have not been characterised for MJF PA11. The material models applied to MJF polyamides are calibrated and validated under a single load case, and none of them represents tension–compression asymmetry. Calibrated models are nonetheless applied to stress states and structural scales beyond those of their calibration. For MJF PA11, the conditions under which a given class of calibrated model remains valid have not been systematically reported. Therefore, the objectives of this study are, for the quasi-static regime: (i) to characterise the tensile mechanical properties of MJF-fabricated PA11 in the rotated print orientations and its compressive properties across the conventional series; (ii) to establish criteria for the selection of calibrated native and asymmetric plasticity material models across stress states and the transfer from coupon to structure; and (iii) to propose material models suitable for defined simulation conditions.

## 2. Materials and Methods

### 2.1. Materials

PA11 powder (commercial name: HP 3D High Reusability PA11) was provided by HP Inc. (Palo Alto, CA, USA), and it had the following properties: particle size of 54 μm, melting point of 202 °C, and print density of 1.05 g/cm^3^.

### 2.2. Test Specimen Preparation

All test specimens were fabricated using an HP Multi Jet Fusion 3D 4200 printer (HP Inc., Palo Alto, CA, USA). The available build volume of the printer was 380 mm × 284 mm × 380 mm. The “balanced” print mode, designed to provide a compromise between look and feel, dimensional accuracy and mechanical properties, was used. All parts were centred in the build volume, with a space of 10 to 20 mm separating each part to allow complete thermal flow. Parts were fabricated using PA11 powder (30% new to 70% reused refresh ratio) sourced from HP, along with their proprietary fusing and detailing agents. After printing, the parts were allowed to cool in the powder bed for 16 h before being unpacked. Loose powder was recycled, and the printed specimens were cleaned with bead blasting and air blasting. Henceforth, the specimens will be referred to as MJF PA11.

**Figure 1:**
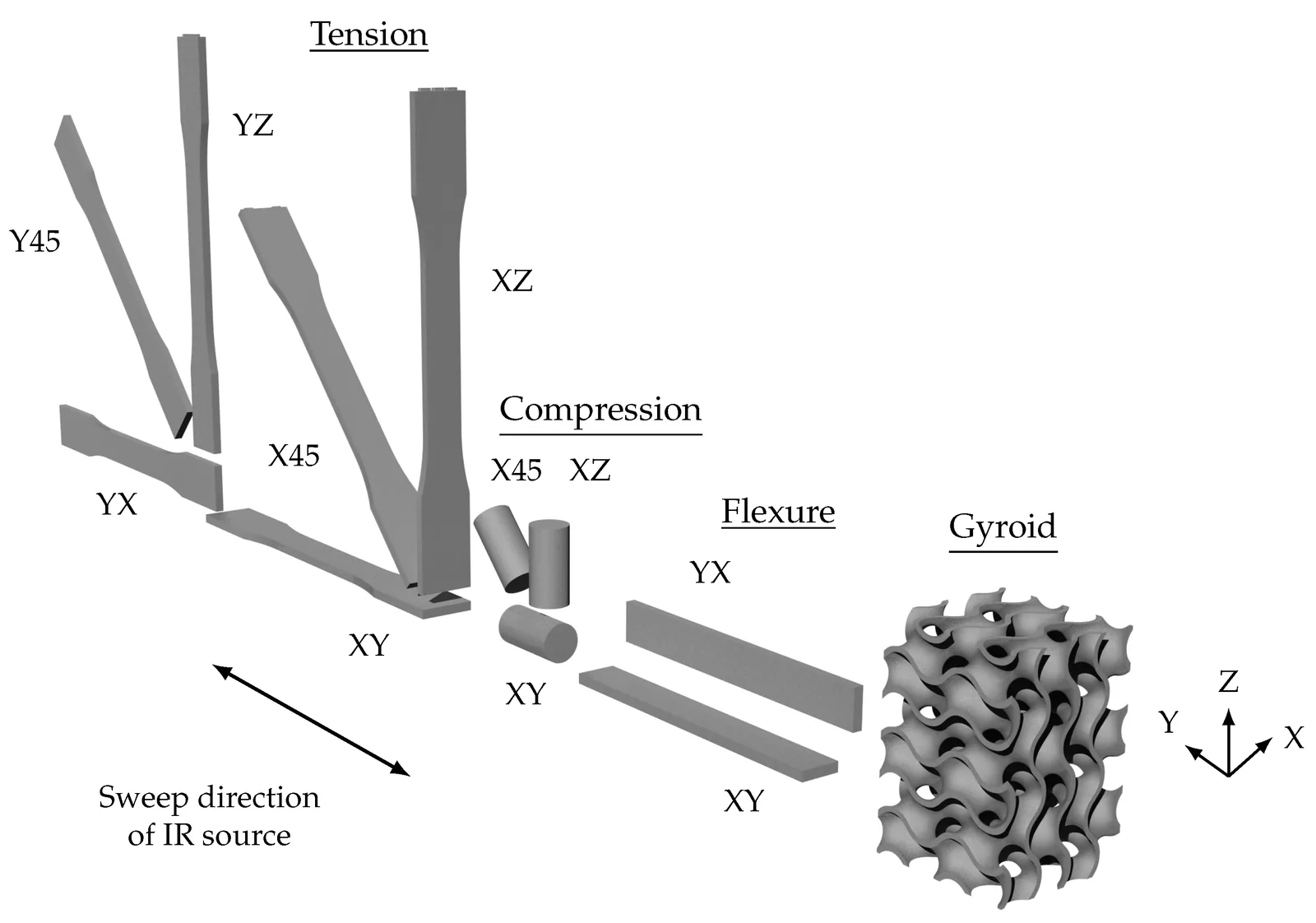
Schematic of the various print orientations with respect to the sweep direction of the IR source.

The characterisation specimens and their respective designations according to print orientation are presented in Figure 1. The length of horizontal specimens (X and Y), and therefore the direction of the tensile and compressive testing loads, were aligned to the sweep direction of the IR source. Compression test specimens had a cylindrical cross-section. Therefore, they were identical when printed in the X and Y directions. The uniaxial gyroid specimen was aligned with the vertical build axis.

### 2.3. Experimental Characterisation Techniques

This section describes the four quasi-static test modes used in the study: uniaxial tension and compression, which provide the calibration data, and three-point bending flexure and gyroid crushing, which serve as independent validation cases. Mechanical property testing for uniaxial tension, uniaxial compression and three-point bending flexure was conducted with an Instron Universal Test Instrument, Model 5900R (Instron, Norwood, MA, USA), with a 30 kN load cell. Uniaxial gyroid crushing was performed with an Instron Universal Test Instrument, Model 5969 (Instron, Norwood, MA, USA), with a 50 kN load cell. Reported property symbols follow the conventions of ISO 527-1 [26]: tensile modulus $E_{t}$, yield strength $\sigma_{y}$, ultimate (maximum) strength $\sigma_{m}$ and strain at break $\varepsilon_{b}$. Compressive and flexural quantities are distinguished by context ($E_{c}$, $\sigma_{f}$, $\varepsilon_{f}$).

Uniaxial tensile testing was performed using Type-I dumbbell-shaped test bars in accordance with ASTM D638-14 [27] at a crosshead speed of 5 mm/min at ambient temperature. Eight specimens were tested for each build orientation (seven for the YZ orientation). Coupons that slipped or failed in the grips were excluded from the reported statistics. Strain was measured with an Instron model XL long-travel extensometer with a gauge length of 50 mm. Yield strength $\sigma_{y}$ was determined at 1% offset, as the pre-yield response of polyamides is markedly nonlinear well below the yield point [28]. $\sigma_{m}$ was taken as the maximum engineering stress and $\varepsilon_{b}$ as the strain at fracture. The raw tension curves carried extensometer noise and a knife-edge seating artefact that produced a non-physical steep foot, in the worst case an apparent secant modulus of 11,400 MPa at 0.1% strain. Both were removed by a scripted pipeline applied identically to every specimen, comprising displacement-binned averaging, isotonic regression for monotonicity, modulus regression over $\sigma \in \left[0.20,0.45\right]\;\sigma_{m}$, and toe compensation after ASTM D638 Annex A1 [27].

Uniaxial compression testing was performed with cylinder specimens of 12.7 mm diameter and 34.925 mm length, giving a slenderness ratio of 11:1 for modulus measurement in accordance with ASTM D695-15 [29], at a crosshead speed of 1.3 mm/min at ambient temperature. Six specimens were tested in the XY orientation and five each in X45 and XZ. The cylindrical cross-section renders the X- and Y-series orientations identical. Compressive strain was computed from crosshead displacement normalised by specimen length. Fixture take-up was removed by toe compensation in accordance with ASTM D695. No load-train compliance calibration was performed, making the reported compressive modulus a lower bound. The compressive modulus $E_{c}$ was determined as the chord from the compensated origin to 2% strain and $\sigma_{y}$ at 1% offset. Because the compressive response densifies monotonically with no load maximum, compressive strength is reported at a fixed reference strain of 10%.

Orientation effects on each tension and compression property were tested by one-way analysis of variance at α=0.05 with Tukey HSD post hoc comparison. Where Levene’s test rejected equal variances, Welch analysis of variance with Games–Howell comparison was used instead. Normality was checked on the standardised residuals by the Shapiro–Wilk test, and the Kruskal–Wallis test was run as a distribution-free check on every omnibus result. Computation used pingouin [30].

Flexural testing was performed with a three-point bending fixture in accordance with ASTM D790-17 procedure A [31], using specimens with a rectangular cross-section of 12.7 mm × 3.2 mm and length 100 mm, providing sufficient overhang for the support span of 51.2 mm. Testing was conducted at an outer-fibre strain rate of 0.01 mm/mm/min, corresponding to a crosshead speed of 1.365 mm/min, at ambient temperature. Three specimens were tested per orientation. Force–displacement curves were toe-compensated per ASTM D790 Annex A1, and flexural stress and strain were computed from the beam-theory reductions $\sigma_{f}=3PL/2bd^{2}$ and $\varepsilon_{f}=6Dd/L^{2}$ up to the 5% flexural strain validity limit.

Uniaxial crushing was performed on a TPMS gyroid structure of size 40 mm × 40 mm × 60 mm with a unit cell size of 20 mm × 20 mm, generated in MSLattice [32] to a design relative density of 20%, which gives a sheet wall thickness of approximately 1.32 mm. Testing was conducted at a crosshead speed of 5 mm/min at ambient temperature in accordance with ASTM D695-15. Test end was set to 45 mm crosshead travel, approximately 75% of the initial specimen height. A single-unit-cell specimen was additionally crushed under the same conditions without platen constraint. This test supports the contact friction sensitivity study described in the finite element methodology.

Across the four test types, the characteristic strain rates span approximately 0.010 to 0.10 mm/mm/min (tension 0.10 mm/mm/min, gyroid crushing 0.083 mm/mm/min globally, compression 0.037 mm/mm/min, flexure 0.010 mm/mm/min). All tests are therefore quasi-static and lie within one order of magnitude of each other, which defines the regime this study addresses. Behaviour at elevated strain rates is outside this scope.

### 2.4. Finite Element Analysis

#### 2.4.1. Material Models and Calibration

The native Abaqus models selected were the EPI and EPC, to which a tension–compression asymmetric yield model was added. Rate-dependent user-material models from the PolyUMod library were also calibrated and tested, but their rate dependence proved incompatible with the velocity scaling that an explicit structural crush requires at practical cost (Section 2.4.3), so they are not carried into the results. The constitutive equations of the native models are reviewed in the literature [33] and are not covered here. The EPI model was included despite the EPC model having previously performed better for MJF PA11 [19], to test its behaviour under more complex conditions. Because the symmetric von Mises yield surface shared by both cannot represent a tension–compression yield asymmetry, each was calibrated as three fits: tension-only, compression-only, and combined. The single-branch fits bound the accuracy achievable within their own stress state, and the combined fit measures the compromise a symmetric surface must strike between the two branches.

Both were calibrated in MCalibration [34] against representative tension and compression curves taken from the coupons of Section 2.3. Calibrating against every coupon of an orientation drives the fit towards its mean response. The coupon closest to that mean was therefore used for each orientation and branch, so that the models are calibrated and compared against a measured curve rather than a constructed one. Parameters were fitted by minimising MCalibration’s normalised mean absolute deviation (NMAD), the mean absolute difference between model and experiment normalised by the mean absolute experimental response. Optimisation ran by random search and NEWUOA until convergence, then by Nelder–Mead simplex refinement of the converged parameter set. No manual post-calibration adjustment of any parameter was performed. For the single-branch fits, the registered chord modulus of the processed experimental curve equals the initial slope of the curve exported to the optimiser by construction. For the combined fits the optimiser is free to adjust the elastic modulus to balance the two branches, and the resulting modulus can depart from the tension chord, which is reported in Section 3.3.

The asymmetric model comprises a Rankine (maximum principal stress) yield surface in tension and a von Mises surface in compression with independent hardening tables, implemented through the cast iron plasticity model native to Abaqus [22]. Although formulated for grey cast iron, it is employed here as a phenomenological carrier of two independently measured flow curves, with no claim that its yield surfaces represent the failure micromechanics of PA11. The conventional alternative is the Drucker–Prager family, where the friction angle sets a single compressive-to-tensile yield ratio. As characterised, that ratio varies with orientation and in some orientations lies below unity. A linear Drucker–Prager surface cannot go below unity. Convexity requires 0<K≤1, so every admissible friction angle gives a ratio of at least one. The cast iron implementation takes the two measured curves as independent tabular inputs and imposes no ratio.

The elastic modulus was taken as the registered chord modulus of the representative tension curve. Neither the native models nor the cast iron model admits a second elastic constant, so no card in this study carries the measured modulus asymmetry (Section 3.1), and the asymmetric model is asymmetric in plasticity alone. Plastic flow in tension is non-associated, with an assumed plastic Poisson’s ratio of 0.3. The tension and compression hardening tables were built directly from the representative curves converted to true stress and plastic strain, with the compression table extended over the measured densification range. The calibrated model was then verified by re-simulating the uniaxial experiments, with no adjustment of the tabulated input.

The formulation carries limitations that bound its use for PA11. The Rankine tension surface depends only on the maximum principal stress, so a second tensile principal stress does not raise the yield point and tension-led multiaxial states may be under-predicted. The compression surface is von Mises with associated flow, and there is no pressure-sensitive transition between the two surfaces. The model carries no rate dependence and no damage. The validation cases of this study, three-point bending and the wall bending and buckling of the gyroid, are dominated by near-uniaxial stress states, so these limitations are not exercised by the results reported here.

Agreement is reported throughout the results as NMAD between the simulated and experimental curves over the comparison range, the mean absolute deviation normalised by the larger of the two mean absolute responses. Values are reported alongside the experimental coupon scatter they compete against, and models within two percentage points of one another are not distinguished, as that separation is smaller than the coupon-to-coupon scatter. Where a calibrated model deviates from experiment in validation, that deviation is reported as a finding rather than tuned away.

#### 2.4.2. Verification and Validation Simulations

**Figure 2:**
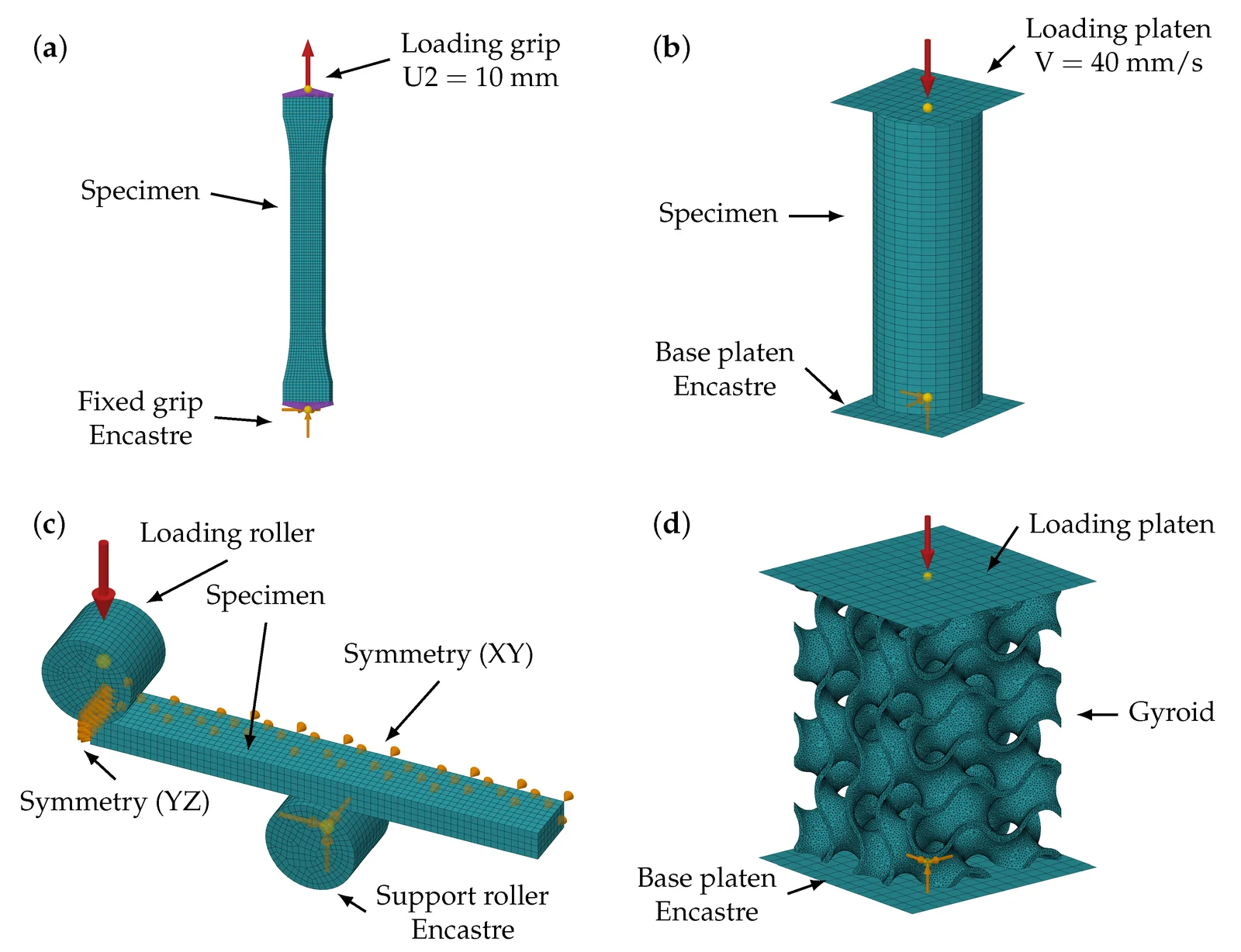
FEA models: (**a**) uniaxial tension, (**b**) uniaxial compression, (**c**) three-point bending, (**d**) gyroid crushing. Red arrows indicate the loading direction.

Every calibrated model was evaluated in three stages of increasing complexity (Figure 2): verification against the uniaxial calibration experiments, cross-validation in three-point bending, where the two branches act simultaneously across the section, and the structural case study of the following section. The verification stage is not an identity check. For the symmetric families it quantifies the deviation of each fit in the opposing stress state, and for the asymmetric model it tests whether one card reproduces both measured branches. Contact is present in three of the four models, in the compression platens, the flexure rollers, and the gyroid platens and wall self-contact. All were modelled with a friction coefficient of 0.13, the value reported for MJF PA11 [10], which was tested rather than adopted directly because it has not been measured for either contact pair present here. A sensitivity sweep on a single-unit-cell model (set up as in Section 2.4.3) at μ∈{0,0.1,0.2,0.3,0.4} found the collapse plateau insensitive to friction above μ≈0.1, supporting the adopted value. The same coefficient is applied throughout.

The tension model replicated the Type-I dumbbell as a 3D deformable solid (Figure 2a), meshed with 4452 C3D8R elements (6420 nodes). Two solver variants share this mesh, a static/implicit analysis with displacement-controlled loading and a dynamic/explicit analysis in which the grip reference point is driven at 20 mm/s through a smooth-step ramp with variable mass scaling. The explicit variant was included because models with near-flat post-yield hardening, the tension-only fits and the asymmetric model, fail implicit convergence shortly beyond the yield knee where explicit integration proceeds through it. Both were run where possible and produce indistinguishable pre-failure responses. All explicit coupon analyses were verified quasi-static by limiting the kinetic-to-internal energy ratio to below 5%.

The compression model replicated the cylindrical specimen between two discrete rigid platens (Figure 2b) as a dynamic/explicit analysis of 2640 C3D8R elements, with general contact, hard pressure–overclosure, and the upper platen driven at 40 mm/s through a smooth-step ramp with variable mass scaling. Engineering stress and strain in both uniaxial models were extracted from the gauge region and converted from the true stress and logarithmic strain reported by Abaqus, so the simulation and experiment are compared in identical measures.

The three-point bending validation was a static/implicit simulation of the flexure test (Figure 2c), with the specimen modelled as a 3D deformable solid in C3D8 elements. Double symmetry reduced the model to one quarter, with symmetry conditions across the XY (ZSYMM) and YZ (XSYMM) planes. A global seed size of 0.75 mm gave 2144 elements, determined by a mesh sensitivity study that defined convergence as a change in maximum principal stress below 1%. The 10 mm diameter support and loading rollers were discrete rigid bodies in R3D4 elements, the supports encastred and the loading roller displaced 6.83 mm, corresponding to the 5% flexural strain limit. Flexural stress and strain were computed from the loading-roller reaction force and displacement through the same ASTM D790 beam-theory reduction applied to the experimental data, so the experiment and simulation pass through a single extraction pipeline.

#### 2.4.3. Case Study

The best performing native models were evaluated under more complex conditions in a TPMS gyroid crushing simulation carried into densification (Figure 2d). The as-designed surface was volume-meshed with the open-source fTetWild library [35]. An ideal edge length of 0.58 mm gave 617,566 C3D10M quadratic tetrahedral elements (998,591 nodes), confirmed by a mesh sensitivity study against two coarser meshes of 302,739 and 208,496 elements (ideal edge lengths 0.73 mm and 0.87 mm), which defined convergence as a change below 2% in plateau force and in force at 45 mm of crush. The mesh was analysed as a 3D deformable solid, placed between two discrete rigid platens with the base encastred and the loading platen driven downward. General contact was defined between all exterior surfaces. Loading followed four stages mirroring the physical crush: contact initiation, a smooth-step ramp of platen velocity, the main crushing travel at constant velocity, and terminal densification. The analysis was dynamic/explicit, the standard approach for lattice crushing, where material nonlinearity, geometric instability and evolving self-contact make an implicit solution impractical [20]. Quasi-static conditions at practical cost were achieved by velocity scaling with variable mass scaling, the platen velocity ramping from 0.5 mm/s to 7 mm/s through the elastic response and first peak, 7 mm/s to 20 mm/s across the plateau, and 20 mm/s to 5 mm/s into densification. The platen therefore travels faster than the experimental crosshead, with the analysis verified quasi-static by the energy balance, the kinetic-to-internal energy ratio remaining below 5% across the loaded history of both full-structure runs. Because velocity scaling changes the simulated deformation rate, only rate-independent models are formally valid in this regime. Both runs remained quasi-static to the experimental end of travel, so densification is retained in the quantitative comparison.

## 3. Results and Discussion

This section presents the results of the experimental characterisation and of the finite element studies built on it. Section 3.1 reports the measured mechanical properties and Section 3.2 the choice of calibration basis that follows from them. The simulation studies are then ordered by increasing complexity: verification simulations of the uniaxial calibration experiments (Section 3.3), a three-point bending case study (Section 3.4), and a gyroid crushing case study (Section 3.5). The models recommended on that basis are given in Section 3.6.

### 3.1. Mechanical Properties

**Figure 3:**
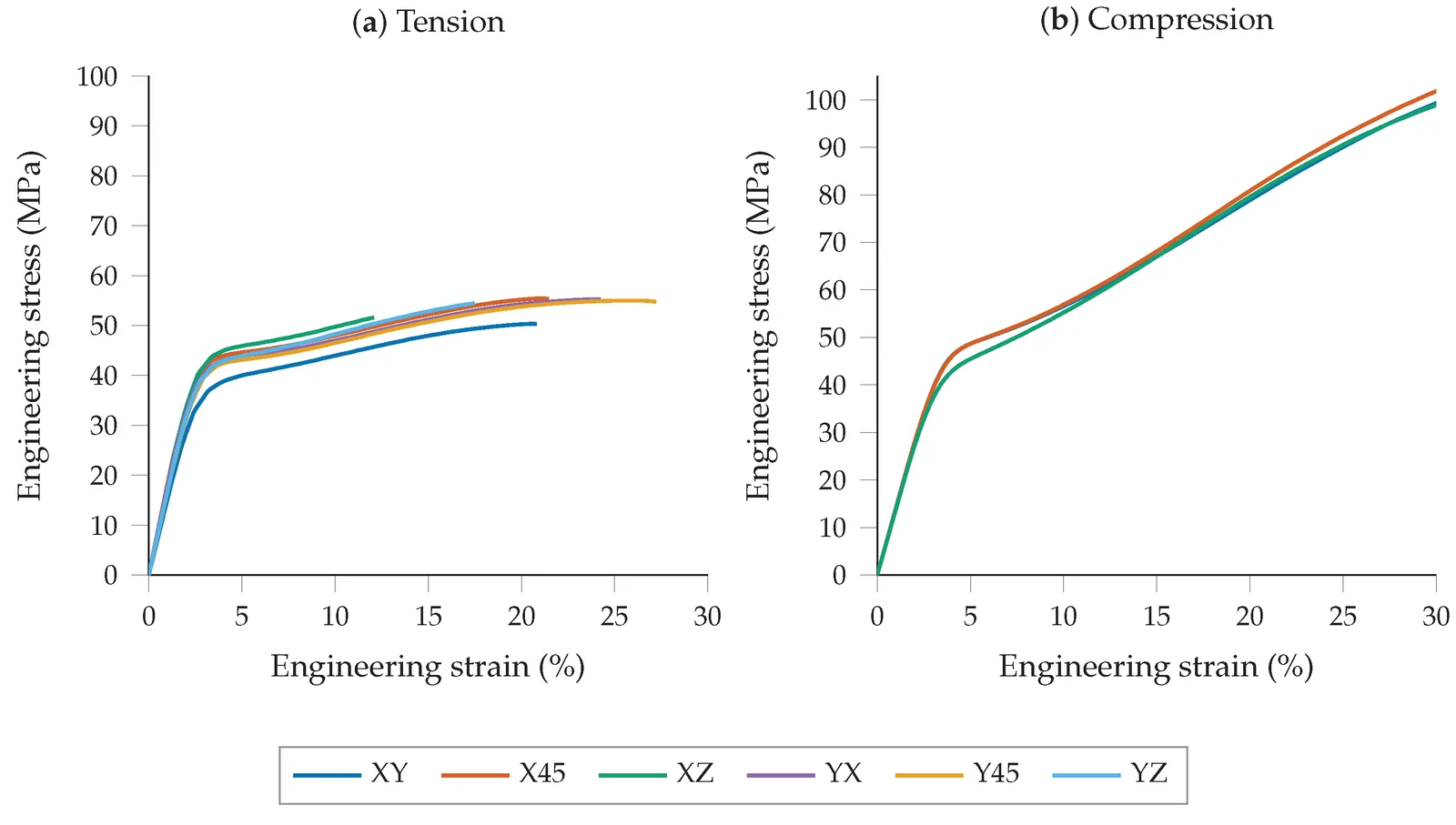
Representative engineering stress–strain response of MJF PA11 by print orientation, showing (**a**) tension in six orientations and (**b**) compression in three orientations (XY, X45, XZ). One representative coupon is shown per orientation, selected as the included coupon closest to its orientation mean. Colours are shared across panels.

**Figure 4:**
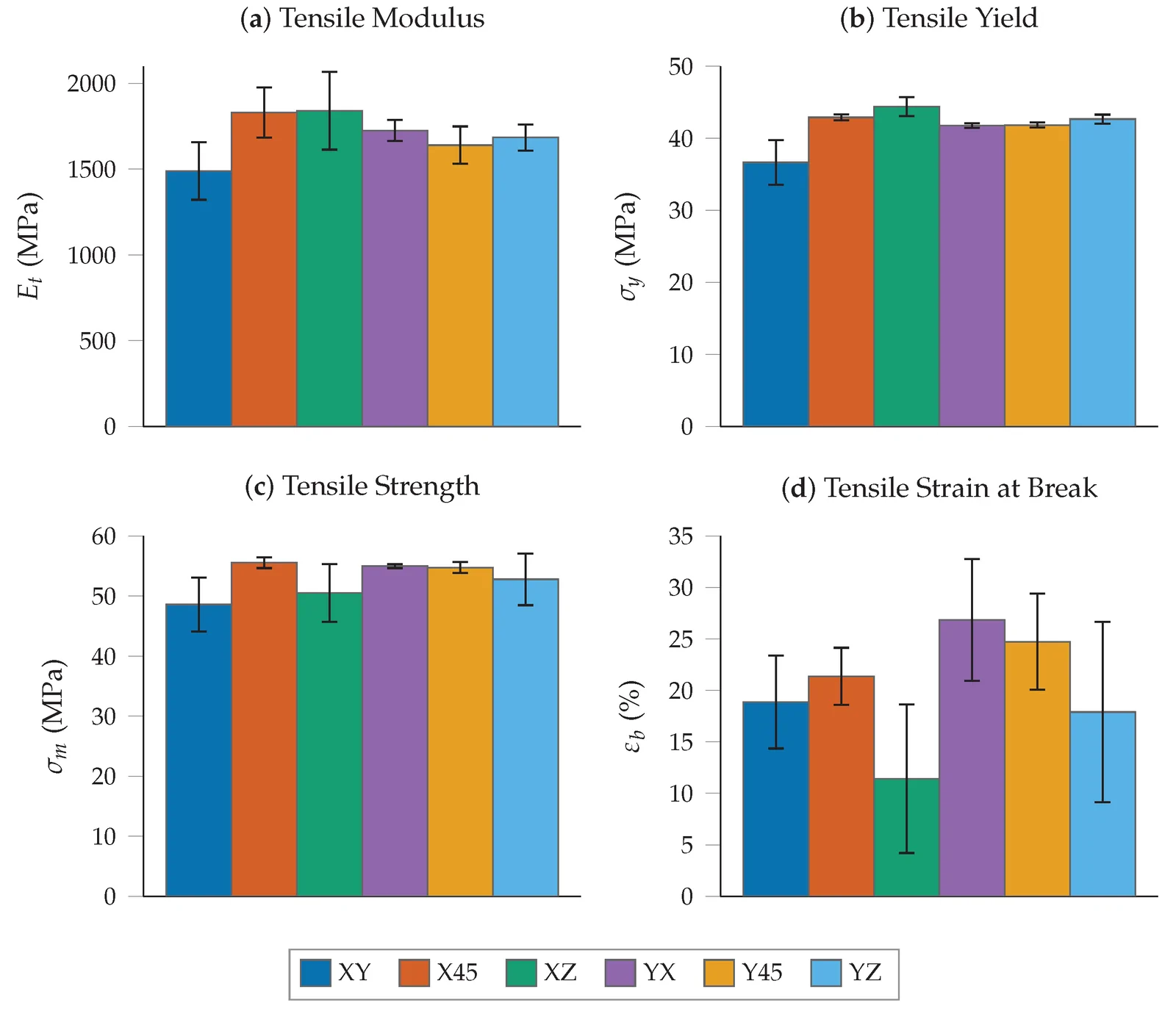
Tensile mechanical properties of MJF PA11 by print orientation. Bars are means of the included coupons and error bars are one standard deviation.

**Figure 5:**
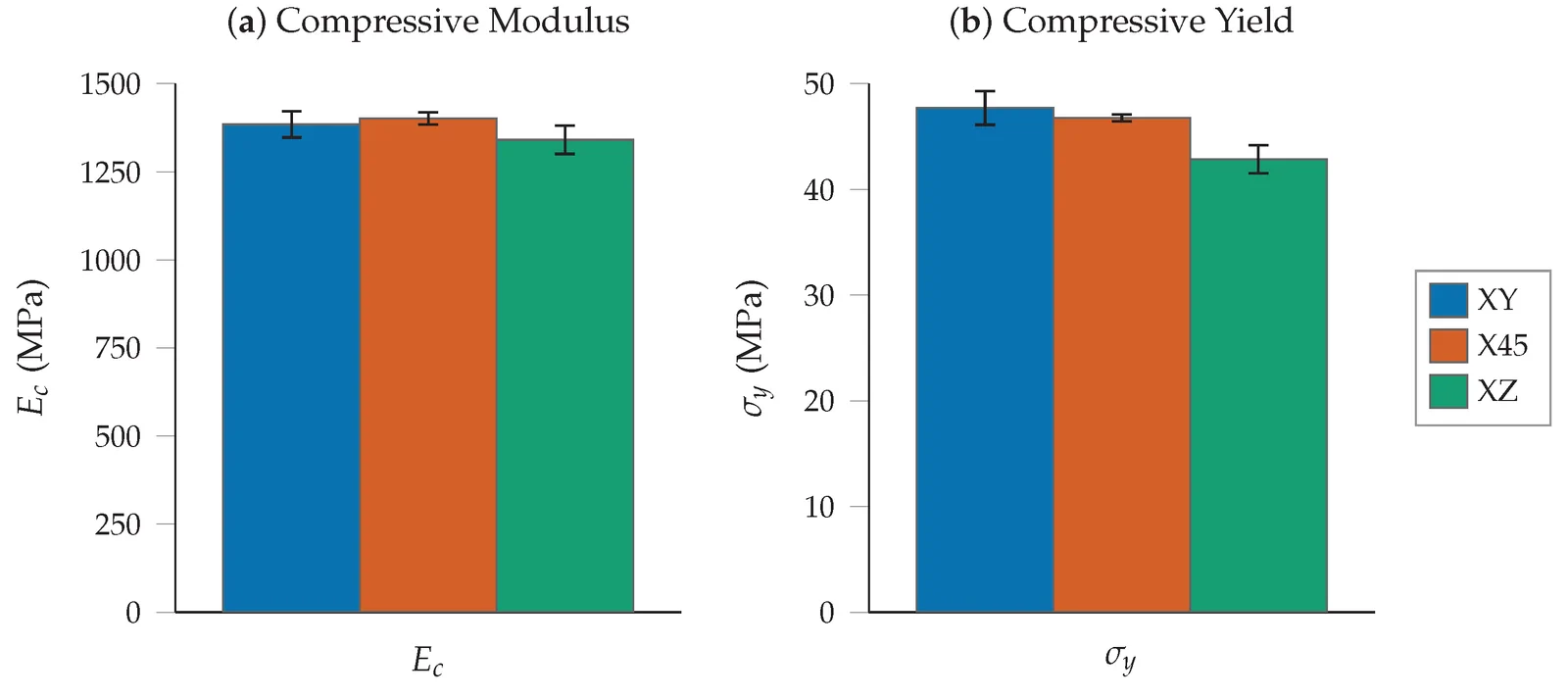
Compressive mechanical properties of MJF PA11 by print orientation. Bars are means of the included coupons and error bars are one standard deviation.

MJF PA11 was evaluated in tension and compression, with representative stress–strain curves in Figure 3 and the derived properties in Figure 4 and Figure 5. Anisotropy appeared in both stress states and was most pronounced in tension among the conventionally oriented prints (XY, X45, XZ), and worst for XY. Differences between the best and poorest orientations were 19.1% for $E_{t}$, 17.4% for $\sigma_{y}$, 12.6% for $\sigma_{m}$ and 46.6% for $\varepsilon_{b}$. XZ and X45 were the stiffest and not significantly different (p=1.000), while XY was the most compliant, significantly below X45, XZ and YX (p≤0.027). XZ carried the highest mean stiffness and yield strength, neither significantly above X45 (p≥0.212), but failed at the lowest elongation (mean 11.42%), significantly below YX, Y45 and X45 (p≤0.036). As reported for PA12 [18], the specimens rotated 90° about the IR-sweep axis (YX, Y45, YZ) were far less anisotropic, with spreads of 4.9% for $E_{t}$, 2.1% for $\sigma_{y}$ and 4.0% for $\sigma_{m}$. No pair within the Y-series differed significantly on any tensile property (p≥0.058). Elongation at break carried the widest scatter, yet orientation still had a significant effect (p<0.001). XZ was the only orientation set apart, and no other pair was resolved (p≥0.061).

In compression the differences between orientations were smaller than in tension, in line with the literature [13], at 4.3% for $E_{c}$ and 10.2% for compressive yield strength. X45 had the highest $E_{c}$ and XZ the lowest, the only pair that differed significantly (p=0.032), with XY not separable from either (Figure 5). In compressive yield strength XY and X45 were not significantly different (p=0.448) and XZ was significantly weaker than both (p<0.001). Every orientation densified monotonically without failure.

Within a single orientation, the two stress states differ. The compressive yield strength exceeds the tensile by a margin that depends on orientation. At XY the margin is 30.2% (47.7 against 36.6 MPa), at YX 14.3%, smaller at X45 and negligible at XZ. The asymmetry is not confined to yield, and the two measures run in opposite directions. The compressive modulus lies below the tensile modulus at every orientation, by 7% at XY and by 20% to 27% at YX, X45 and XZ. These margins are upper bounds, as the compressive modulus was derived from crosshead displacement without a load-train compliance calibration (Section 2.3). XY carries the largest yield asymmetry and the smallest modulus asymmetry, and XZ the reverse. A model symmetric in tension and compression cannot reproduce both branches of the XY response at once, which is examined in Section 3.3. The anisotropy in MJF has been linked to porosity and IR exposure time [13,14,18]. As in PA12, tensile properties improved as specimens were oriented towards the vertical build direction, and the best overall performance came from specimens built at a 45° inclination to the build plate, consistent with published work [13]. Those specimens were not significantly different from XZ in tensile modulus or yield strength (p≥0.212) and broke at a significantly higher strain (p=0.036). The anisotropy extremes are the two ends of the conventional series, the flat XY and vertical XZ orientations, so orientation matters most for slender components aligned with one of them. Given the reduced anisotropy of the Y-series, slender components should be oriented with the smallest dimension parallel to the XY plane, which increases IR exposure. Where elongation at break governs the design, XZ warrants caution, as it failed at a significantly lower strain than YX, Y45 and X45 despite its stiffness and strength.

### 3.2. Choice of Calibration Basis

Two calibration bases, XY and YX, were carried through the study. YX is the primary basis and is proposed for general components. It sits within the near-isotropic cluster of Section 3.1, where the Y-series tensile spreads are 4.9% for $E_{t}$ and 2.1% for $\sigma_{y}$. YX is not significantly different from any other non-XY orientation in tensile modulus (p≥0.614), and in yield strength it is not separable from Y45 or YZ (p≥0.058). It lies 2.7% below X45 and 5.9% below XZ in yield strength (p≤0.026), so a YX calibration is marginally conservative for those orientations. Every model evaluated here is isotropic, so YX is a choice of calibration data rather than a claim about the material. The choice does not extend to slender components whose smallest dimension lies in the build direction (XY orientation). The compression result is shared between the bases, as the cylindrical specimen is geometrically identical for the X- and Y-series (Section 2.2). The two families are referred to hereafter as the YX-calibrated and XY-calibrated models.

XY was retained as the demanding case. It carries the largest tension–compression yield asymmetry of the orientations characterised in both stress states, approximately 30.2% against 14.3% at YX, so it is the most severe test of the symmetric yield surface assumption. It is also the anisotropy extreme, with the lowest tensile modulus and yield strength, an outlier rather than a representative state of the material. Its depressed tensile response is associated with the flat build geometry of the coupon, in which the smallest specimen dimension lies in the build direction and few layers span the thickness. A model calibrated to XY therefore under-predicts the stiffness and strength of the near-isotropic majority by up to the X-series spreads of 19.1% in $E_{t}$ and 17.4% in $\sigma_{y}$. The consequences of the symmetry assumption are examined in Section 3.3, and a further orientation-specific limitation in bending in Section 3.4.

### 3.3. Verification of the Calibrated Material Models

**Figure 6:**
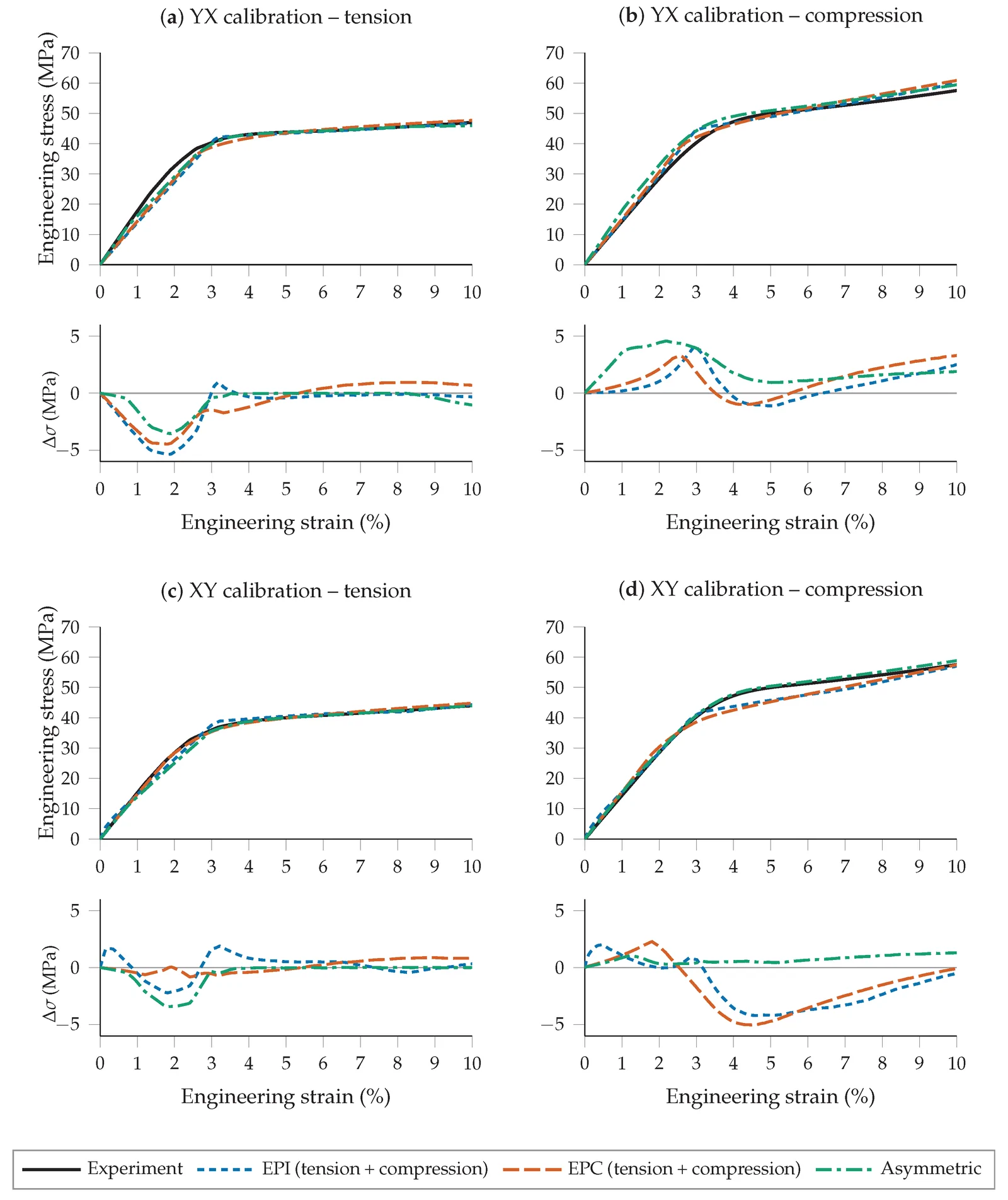
Verification simulations of the three material models that carry both uniaxial branches, compared against their calibration curves (the specimens closest to the orientation means, Section 2.3). The primary YX basis is shown in (**a**) tension and (**b**) compression, and the XY basis in (**c**) tension and (**d**) compression. The compression specimen is cylindrical and therefore common to both bases, so panels (**b**,**d**) share the same experimental curve. The strip beneath each panel shows the residual, simulation minus experiment, over the same strain window.

Fit quality was assessed through the verification simulations of the uniaxial calibration experiments described in Section 2.4.2, comparing each model against its representative specimen. The XY and YX models were compared against a shared compression specimen. Calibration completed without manual adjustment for every model. The primary YX basis is presented first, followed by the XY basis. Deviations are quantified as NMAD between the engineering stress–strain curves, in which the experimental curve is interpolated at the simulation increments and the mean absolute deviation is normalised by the larger of the two mean absolute stresses. The comparison window is 0% to 10% engineering strain in both stress states, which is the range plotted in Figure 6. Every card is compared over this window, except for the EPI tension-only fit in compression, whose analysis terminated before the window was reached.

Over the strain window, each single-branch fit was most accurate in the stress state it was calibrated to and less accurate in the other (Table 1). The tension-only fits held tension to 0.5% (EPI) and 1.3% (EPC), and the compression-only fits mirrored them, holding compression to 2.1% and 2.2% while deviating by 6.1% and 4.9% in tension. Only the EPI tension-only card failed outright in the opposing branch. The EPC tension-only fit was the least reliable card of the family in compression at 5.1%, and the EPI tension-only analysis terminated by element distortion under the platen at approximately 0.7% nominal strain, before any usable prediction was produced. The models that carry both tension and compression calibration compromise between the two, and at this basis the compromise is not considerable (Figure 6a,b). Over the same window the asymmetric model was the most accurate of the three in tension, at 2.1%, and the least accurate in compression, at 4.9%. The combined fits traded in the opposite direction, the EPI fit deviating by 4.0% in tension and 2.5% in compression and the EPC fit by 4.3% and 3.4%. None of the three exceeded 5% in either branch. The compromise is more visible at the XY basis, where the tension–compression asymmetry is larger.

The XY basis carries the larger tension–compression asymmetry, so the cost of the symmetry assumption is clearest there. In tension (Figure 6c), every model informed by the tension branch performed similarly. Over the 0% to 10% window the combined fits deviated by 2.5% (EPI) and 1.4% (EPC) NMAD, and the asymmetric model by 2.0%. The tension-only fits were closer still, at 1.4% and 1.1%. The compression-only fits, which saw no tension data, deviated by 6.9% and 7.8% and stayed stiffer than the experiment through the yield region and along the plateau. In the combined fits the error sits at the yield knee rather than on the plateau, which both track to within approximately 2%, moving from slight under-prediction near 4% strain to slight over-prediction by 10% strain. In compression (Figure 6d) the order reverses. The asymmetric model was closest at 1.7%, and the compression-only fits followed at 2.1% (EPI) and 2.2% (EPC). The combined fits deviated by 5.1% and 5.2%, under-predicting the early plateau because the calibration adopted a yield stress near the tensile value for both branches. The tension-only fits were the least accurate at 6.2% and 6.5%.

Taken together, the verification simulations separate what each calibration choice delivers from what it costs. The models carrying both branches worked, with deviations between 1.4% and 5.2% across both stress states and both bases. The asymmetric model reached 2.1% in tension at YX, though it was the least accurate of the three in compression there at 4.9%, and it carried the XY branches at 2.0% and 1.7%, the only card of that family below 2.5% in each. The combined EPC fit was the best of the symmetric natives, in agreement with the earlier study of this material [19]. Calibration to a single stress state comes at a cost. Each single-branch fit was accurate in its own branch and materially worse in the other, and the tabular EPI tension-only fit failed outright in compression at the YX basis. The EPI fits hold the flow curve as a table that Abaqus extrapolates as perfectly plastic, so once the material under the platen exceeds the tabulated range it carries zero tangent modulus and localises until the elements distort, whereas the parametric EPC law retains a positive tangent modulus and merely loses accuracy. The combined fits reconcile the two branches by moving the elastic modulus off the measured tension chord, and the move is towards the measured compressive modulus. At YX, where the two differ by 20%, the EPI and EPC combined fits adopted 1407 MPa and 1459 MPa against a tension chord of 1735 MPa and a compressive modulus of 1384 MPa. At XY, where they differ by 7%, the EPC combined fit adopted 1493 MPa and barely moved. The size of the trade tracks the measured modulus asymmetry. The EPI combined fit at XY is the exception, adopting 2636 MPa, above both measured moduli. A card whose elastic modulus exceeds both measured branches is not an admissible representation of the material, and in bending that inflated modulus would recover the measured stiffness for the wrong reason. Consequences of the modulus trade for bending are examined in Section 3.4. The asymmetric model avoids that trade because its two hardening tables are independent inputs, and pays instead through the Rankine yield surface, which under-runs the experiment between approximately 1% and 4% strain and, with near-flat post-yield hardening, forces the coupon analyses into the explicit solver. The EPI family is not carried further, on the truncation failure of its tabular fits and the inadmissible modulus of its XY combined fit. The EPC combined and asymmetric models proceed to the case studies that follow, where they are tested against data that took no part in their calibration.

### 3.4. Case Study: Three-Point Bending

**Figure 7:**
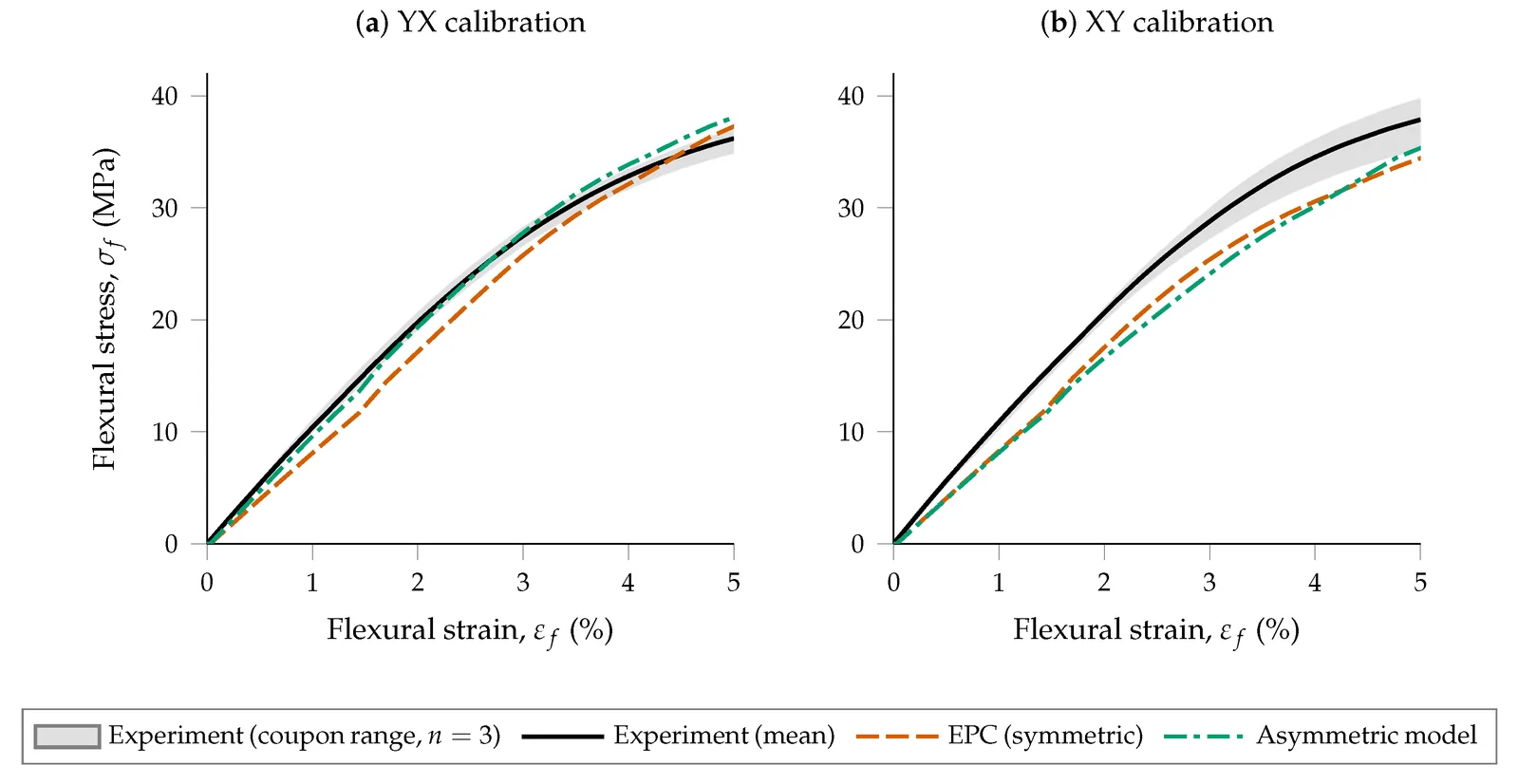
Three-point bending cross-validation of the combined symmetric (EPC) and asymmetric (cast iron) models against the toe-compensated procedure-A flexure experiment of the matching orientation, for (**a**) the YX and (**b**) the XY calibration basis. The shaded band is the pointwise range of the three coupons.

Three-point bending was used as the first case study because it engages both uniaxial branches simultaneously across the section, with the comparison conducted up to the 5% flexural strain limit (per Note 5, ASTM D790) [31]. The comparison was made at the material level. Experimental stresses were computed from each specimen and from the simulation through identical beam-theory reduction, so specimen-to-specimen geometry variation is normalised out. Elastic-range comparisons use the slope fitted up to 1% flexural strain, in accordance with ASTM D790 and applied identically to the experiment and simulation. The two bases are compared against their own flexure experiments in Figure 7.

The two material cards carried into the case studies performed similarly in both uniaxial branches. Bending, however, separates them (Figure 7a). The asymmetric model, which retains the measured tension chord, tracked the YX coupon well with 3.9% NMAD. The combined EPC fit sat below the coupon envelope over most of the range, at 8.2% NMAD, and its elastic slope fell 21.8% below the measured slope against 7.1% for the asymmetric model. That separation is the modulus trade of Section 3.3 surfacing as a prediction error, and it is a property of the compromised modulus fit. The XY family behaves differently (Figure 7b). The XY-calibrated models under-predicted the measured flexural response, by 14.1% (EPC) and 16.2% (asymmetric) NMAD against a coupon scatter band of −7.1 to +5.0 percent, with fitted slopes 23.4% and 24.5% below the measured slope and the deficit narrowing as strain increases. The deficit is common to both models, including the asymmetric card that carries the measured chord modulus at both bases. No combination of the measured tension and compression behaviour evaluated here reproduces the measured bending response.

Possible explanations were examined systematically. Machine compliance can only soften a measured response, and the experiment is stiffer than both models, so it is excluded. Loading rate goes the same way, as flexure is the slowest of the four tests and a rate mismatch would act in the opposite direction. The mesh study, the measured-dimension reduction and the shared beam formula exclude mesh discretisation, specimen geometry and the reduction itself, and a sweep of the elastic Poisson’s ratio moved the reduced stiffness by less than 1%. The same slope construction is applied to the experiment and simulation, so the extraction cannot be the cause. Nor is the yield surface. A linear Drucker–Prager card calibrated against the same test data, pressure-sensitive where the other two are not, under-predicts by 13.8% NMAD. The elevation is not peculiar to this study either, as flexural modulus has been under-estimated before for MJF PA11 [19] and PA12 [18], across three studies and two materials, which is consistent with an origin in the MJF process rather than in one test series. What remains is the contrast between the bases. The asymmetric card carries the measured chord modulus at both, so comparing Figure 7a against Figure 7b isolates orientation from calibration, and the same construction recovers the experiment at YX while falling 24.5% short at XY. The coupons say the same. XY is stiffer in flexure than YX, a chord modulus of 1099 MPa against 1051 MPa over the 1 MPa to 8 MPa stress window, despite a tensile modulus about 13.7% lower. What causes it is unknown. A stiffer near-surface region would produce the elastic signature, and a layered section model that preserves the uniaxial response reproduces it. Bending weights the outer fibres where tension weights the whole section, so a through-thickness gradient acts unequally on the two tests. That shows a gradient could produce the elevation, not that it does. Confirming it needs microstructural measurement.

### 3.5. Case Study: Gyroid Crushing

**Figure 8:**
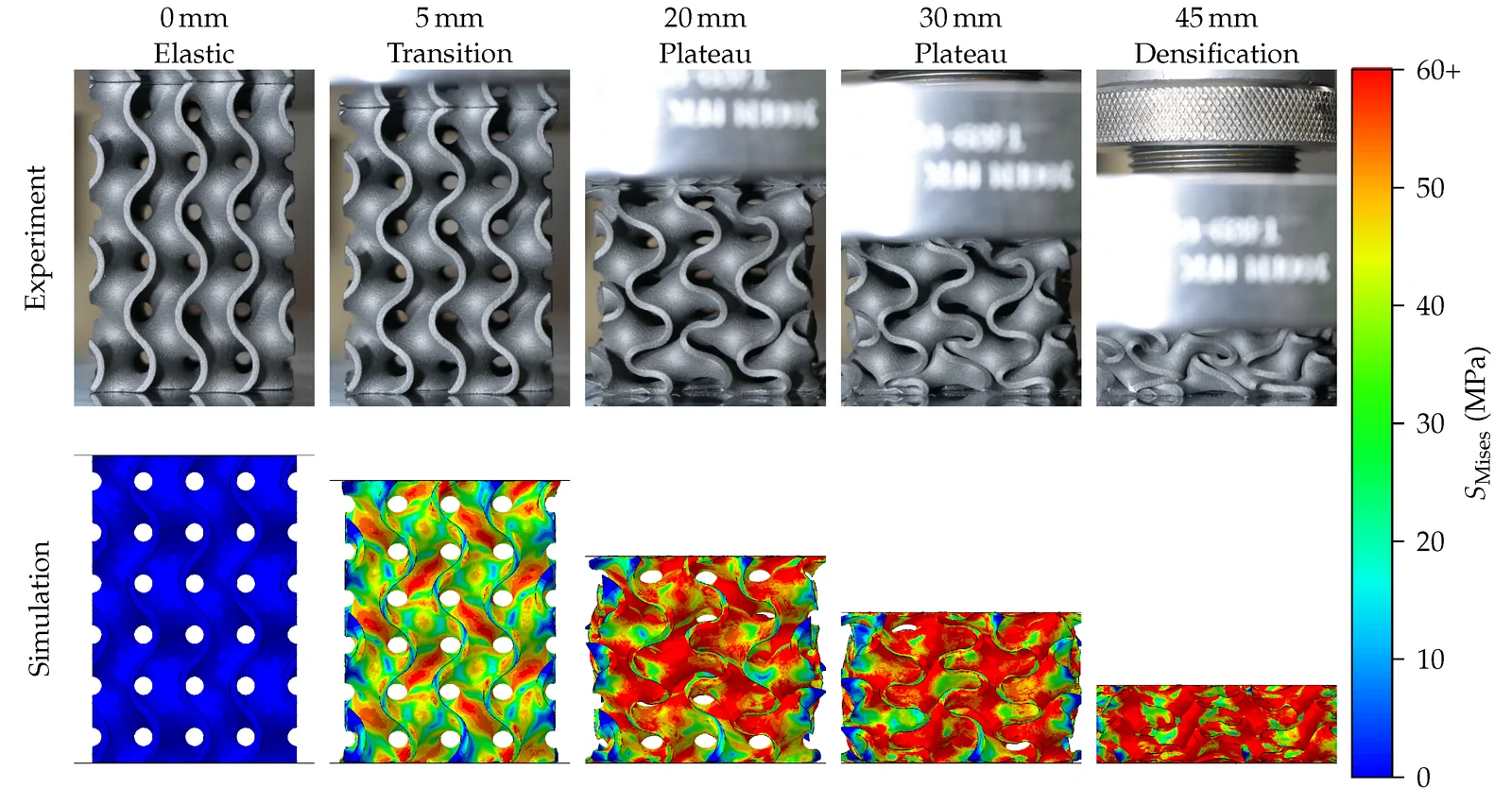
Experimental and simulated crushing of the TPMS gyroid specimen at matched plate displacements through the elastic, plateau and densification phases, the 5 mm frame marking the elastic-to-plateau transition. Simulation frames show the von Mises stress field of the combined-fit EPC model between the rigid platens. Stress above 60 MPa (≈1.5 times the material yield), reached across the densified structure and peaking near 135 MPa in localised wall contact, is clipped to the top of the scale.

**Figure 9:**
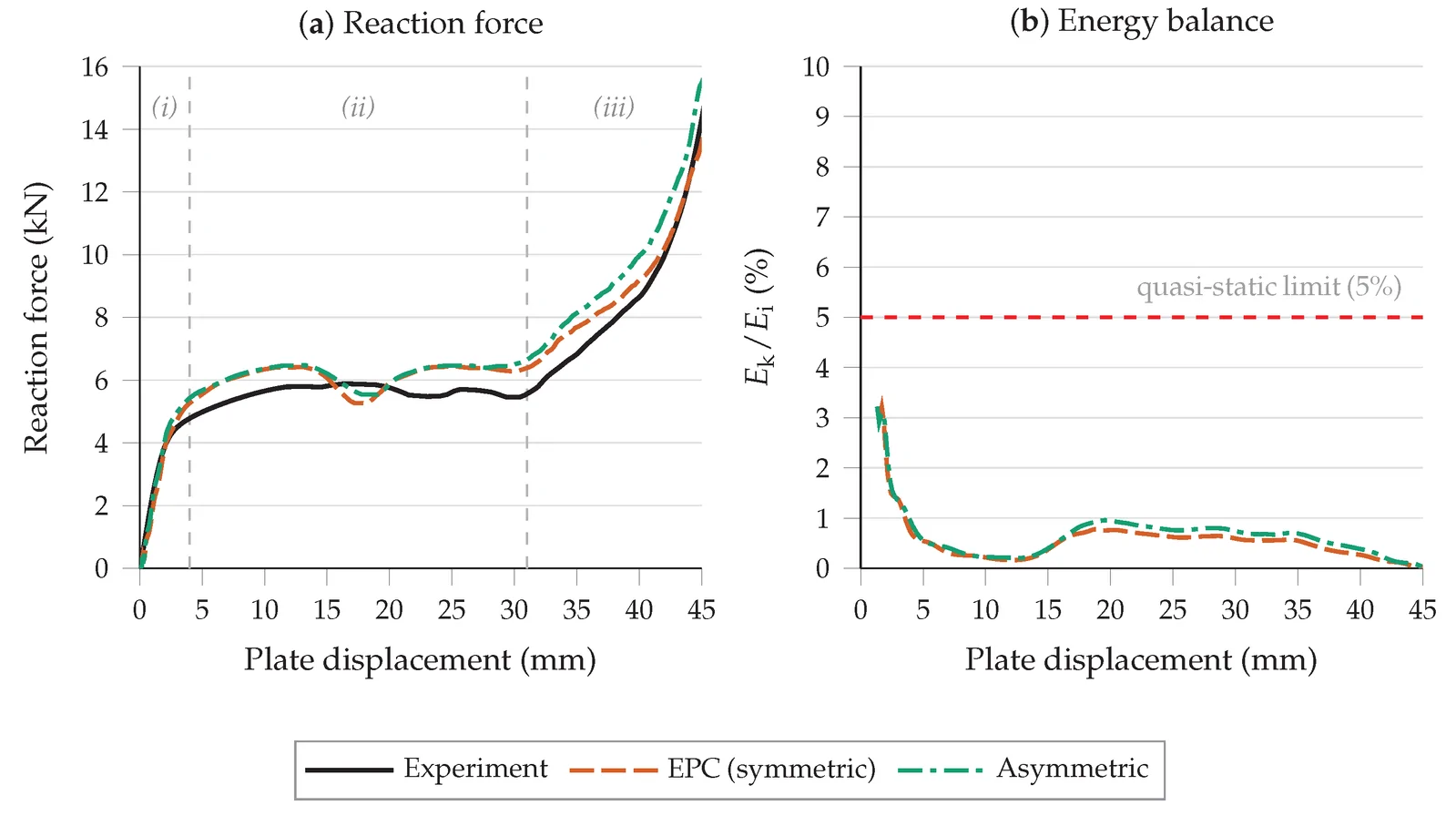
Reaction force against plate displacement for the gyroid crush, comparing the combined-fit EPC and asymmetric material models against the experiment through the (i) elastic, (ii) plateau, and (iii) densification phases. Both models reproduce the crush morphology and over-predict the plateau and densification response, indicating that the measured structure is softer than either coupon-calibrated model predicts.

A TPMS gyroid was crushed between two rigid plates, the first test of the calibrated models on a structure rather than a coupon. The load case is demanding. It engages the full material response through an elastic phase, a plateau of progressive cell collapse, and a densification phase, each governed by different mechanics. The analysis also carries general contact with specimen wall self-contact. Plate travel reaches 45 mm on a 60 mm specimen. Both analyses were confirmed quasi-static, with a kinetic-to-internal energy ratio averaging 0.5% against the 5% limit recommended in the Abaqus documentation. Both reproduced the deformation sequence at matched plate displacements (Figure 8) and followed the measured force–displacement response through all three phases (Figure 9), the EPC model to 8.7% NMAD over the full crush and the asymmetric model to 11.0%. Cards calibrated from uniaxial coupons alone therefore transfer to a structure whose response is governed by buckling, progressive collapse and self-contact.

The residual is a modest one-sided offset. Both models over-predict the plateau, by 9.0% (EPC) and 10.6% (asymmetric) in mean signed deviation. Several features of the analyses act in that direction. The mesh was built from the nominal as-designed surface, so its walls are uniform and carry none of the porosity, surface roughness or local thickness variation a printed part may have, each of which would lower the load at which a wall buckles and yields. The crush is also compression-dominated, and each card carries a single elastic modulus. At the YX basis that modulus sits above the measured compressive modulus by 5.4% for the EPC fit, whose modulus is a compromise between the branches, and by 25.4% for the asymmetric card, which carries the tension chord unchanged. Both models are therefore stiffer than the material in the stress state that governs, the asymmetric card by the larger margin.

The structure is also strongly sensitive to wall thickness. The same gyroid was analysed with a wall thickness of 1.20 mm, 9% below the design. The mean plateau force of the EPC model, a ratio of phase means rather than the pointwise signed deviation quoted above, moved from 8.8% above the measurement to 4.0% below it, and the first peak from 10.6% above to 1.2% below, so a reduction near 0.1 mm would close the plateau offset. The force at 45 mm of crush moved from 5.2% to 17.4% below the measurement, so thickness does not account for the response as a whole. The as-printed wall could only be measured with digital callipers on the exposed edges, giving 1.3 mm to 1.4 mm against the as-designed 1.32 mm, and those edges need not represent the mid-wall. A deficit of the order that matters here can therefore be neither confirmed nor excluded. The single elastic modulus is an established contributor to the residual and the as-printed geometry a plausible one. Neither accounts for it in full, and the plateau predictions should be read as an upper bound on crush resistance.

### 3.6. Recommended Models

The YX basis is recommended for general components. It sits within the near-isotropic cluster of Section 3.1, and both models recommended here held single-digit NMAD in the uniaxial branches (Table 1) and in bending (Section 3.4). The XY basis is not recommended for design. It was carried as the constraining case, and neither XY-calibrated model reproduced the measured bending stiffness. Components should be built away from the flat XY orientation (Section 3.1). Calibration to a single stress state serves only applications dominated by that state, and the tabular EPI tension-only card did not merely lose accuracy in compression but terminated by element distortion at the YX basis (Section 3.3). Fits calibrated to both branches compromise between them, and that compromise is the better trade. Two are recommended, for different reasons.

The EPC combined fit is recommended as the general-purpose card. Its parametric hardening law extrapolates beyond the calibrated range where the tabular fits terminated by truncation, it converges in the implicit solver where the asymmetric model forces explicit integration, and at the YX basis it deviated by 4.3% in tension, 3.4% in compression and 8.2% in flexure. In a compression-dominated crush the two models separated only weakly, so it is also the economical choice at that scale (Section 3.5). The asymmetric model outperformed it in bending because its independent hardening tables let the calibration hold the elastic modulus at the measured tension chord (Section 3.3). That agreement is empirical. The formulation was built for grey cast iron, its yield surfaces do not represent the pressure-dependent shear yielding of PA11, and its calibration here was uniaxial, so its shear and multiaxial response is inherited rather than measured (Section 2.4.1). It is worth considering where bending stiffness governs and the stress state is near-uniaxial, and within those limits only. The EPC combined fit remains the recommendation for general use.

## 4. Conclusions

MJF PA11 was characterised in tension across six build orientations and in compression across three, and a family of finite element material models calibrated from that data was evaluated in the quasi-static regime. Build-direction anisotropy was greater in tension than in compression. Tensile performance improved as specimens were oriented away from the flat XY build, and was best at a 45° inclination. Anisotropy fell markedly in specimens rotated 90° about the IR sweep axis, consistent with increased IR exposure. The flat XY orientation is the anisotropy extreme rather than a representative state. Its compressive yield strength exceeded its tensile yield strength by approximately 30.2%, an asymmetry that fell to approximately 14.3% at the near-isotropic YX orientation and became negligible by XZ. Two calibration bases were therefore carried through the study, a YX basis representative of general components and an XY basis as the constraining case.

A symmetric yield surface cannot carry the measured tension–compression asymmetry. Single-branch fits deviated by up to 7.8% in the opposing stress state, and the tabular tension-only fit was worse than inaccurate in compression at the YX basis, failing by element distortion where its hardening table runs out. The isotropic-hardening model therefore requires both branches over the strain range of the application. Fits calibrated to both branches compromise between them, and the asymmetric plasticity model carrying both measured hardening curves was the only card to hold both branches at the XY basis, though at YX it traded compressive accuracy for tensile. Carried from the coupon to a structure, both leading models reproduced the three-phase crush of a TPMS gyroid to within 11% NMAD, with a modest over-prediction of the collapse plateau, so the predictions are an upper bound on crush resistance.

One departure remains unexplained. In three-point bending the asymmetric model reproduced the YX response within the coupon scatter, while the measured response of the XY coupons exceeded both XY-calibrated models, most strongly in the elastic range. This flexure elevation is specific to the flat XY build. A through-thickness stiffness gradient could produce its elastic signature, but the hypothesis is untested and the cause is open.

These findings hold within the conditions tested. One PA11 powder at a single refresh ratio was printed on one machine in one print mode, with every specimen centred in the build volume. Loading was quasi-static, within one order of magnitude of strain rate across the four tests, and structural validation covers three-point bending and a single gyroid crushing configuration. Each model was calibrated against one representative coupon per branch. The compressive modulus came from crosshead displacement without compliance calibration and is a lower bound. The gyroid was analysed on its nominal surface, which carries none of the porosity, roughness or local thickness variation a printed part may have. Both validation cases are near-uniaxial, so the multiaxial response of the asymmetric yield surface is untested.

Within those bounds, the YX orientation is recommended as the calibration basis for general components. The combined-hardening EPC fit calibrated against both branches is recommended as the general-purpose card, holding single-digit deviation across tension, compression and flexure at that basis. The asymmetric model outperformed it in bending because its tension and compression hardening tables are independent inputs, so the elastic modulus did not have to be compromised between the two branches and was set at the measured tension chord (Section 3.3). That agreement is empirical, the formulation was built for grey cast iron, and it should be considered only where bending of slender sections governs, the near-uniaxial stress state in which it was validated. Further investigation of the flexure elevation by microstructural measurement is warranted, as is the behaviour of the material and of the rate-dependent models at elevated strain rates.

## Figures and Tables

**Table 1:** Verification NMAD values at the YX basis, evaluated against the YX tension and the shared compression curves.

Model	Tension (%)	Compression (%)
EPI tension-only	0.5	terminated ^†^
EPC tension-only	1.3	5.1
EPI compression-only	6.1	2.1
EPC compression-only	4.9	2.2
EPI combined	4.0	2.5
EPC combined	4.3	3.4
Asymmetric	2.1	4.9

^†^ Analysis terminated by excessive element distortion at approximately 0.7% nominal strain.

## Data Availability

The data presented in this study are available from the corresponding author on reasonable request.
